# Abundance of Non-Polarized Lung Macrophages with Poor Phagocytic Function in Chronic Obstructive Pulmonary Disease (COPD)

**DOI:** 10.3390/biomedicines8100398

**Published:** 2020-10-08

**Authors:** Kentaro Akata, Kei Yamasaki, Fernando Sergio Leitao Filho, Chen Xi Yang, Hiroto Takiguchi, Basak Sahin, Beth A. Whalen, Cheng Wei Tony Yang, Janice M. Leung, Don D. Sin, Stephan F. van Eeden

**Affiliations:** 1 Department of Respiratory Medicine, University of Occupational and Environmental Health Japan, Fukuoka 807-8555, Japan; kentarouakata@med.uoeh-u.ac.jp (K.A.); kkyamsaki1019@yahoo.co.jp (K.Y.); 2 Department of Medicine (Respirology), University of British Columbia, Centre for Heart Lung Innovation, St. Paul’s Hospital, Vancouver, BC V6Z1Y6, Canada; fernando.studart@hli.ubc.ca (F.S.L.F.); yolanda.yang@hli.ubc.ca (C.X.Y.); takihiroto@gmail.com (H.T.); basak.sahin@hli.ubc.ca (B.S.); beth.whalen@hli.ubc.ca (B.A.W.); Tony.yang@hli.ubc.ca (C.W.T.Y.); janice.leung@hli.ubc.ca (J.M.L.); don.sin@hli.ubc.ca (D.D.S.)

**Keywords:** macrophages, phagocytosis, COPD, phenotype, inflammation

## Abstract

Lung macrophages are the key immune effector cells in the pathogenesis of Chronic Obstructive Pulmonary Disease (COPD). Several studies have shown an increase in their numbers in bronchoalveolar lavage fluid (BAL) of subjects with COPD compared to controls, suggesting a pathogenic role in disease initiation and progression. Although reduced lung macrophage phagocytic ability has been previously shown in COPD, the relationship between lung macrophages’ phenotypic characteristics and functional properties in COPD is still unclear. (1) Methods: Macrophages harvested from bronchoalveolar lavage (BAL) fluid of subjects with and without COPD (GOLD grades, I–III) were immuno-phenotyped, and their function and gene expression profiles were assessed using targeted assays. (2) Results: BAL macrophages from 18 COPD and 10 (non-COPD) control subjects were evaluated. The majority of macrophages from COPD subjects were non-polarized (negative for both M1 and M2 markers; 77.9%) in contrast to controls (23.9%; *p* < 0.001). The percentages of these non-polarized macrophages strongly correlated with the severity of COPD (*p* = 0.006) and current smoking status (*p* = 0.008). Non-polarized macrophages demonstrated poor phagocytic function in both the control (*p* = 0.02) and COPD (*p* < 0.001) subjects. Non-polarized macrophages demonstrated impaired ability to phagocytose *Staphylococcus aureus* (*p* < 0.001). They also demonstrated reduced gene expression for CD163, CD40, CCL13 and C1QA&B, which are involved in pathogen recognition and processing and showed an increased gene expression for CXCR4, RAF1, amphiregulin and MAP3K5, which are all involved in promoting the inflammatory response. (3) Conclusions: COPD is associated with an abundance of non-polarized airway macrophages that is related to the severity of COPD. These non-polarized macrophages are predominantly responsible for the poor phagocytic capacity of lung macrophages in COPD, having reduced capacity for pathogen recognition and processing. This could be a key risk factor for COPD exacerbation and could contribute to disease progression.

## 1. Introduction

Macrophages are key effector cells in orchestrating both the innate and adaptive immune responses [1,2]. Alveolar macrophages identify, engulf, and process inhaled pathogens, cigarette smoke (CS) and ambient particulate matter (PM) to promote lung homeostasis and prevent overwhelming infections [3]. The number of alveolar macrophages (AM) in bronchoalveolar lavage fluid (BAL) is higher in cigarette smokers and subjects with chronic obstructive pulmonary disease (COPD) compared to nonsmokers [4,5]. Emphysematous destruction, and COPD severity, are related to the accumulation of macrophages in airspaces [6,7], suggesting that macrophages play a pivotal role in orchestrating the chronic inflammatory response in lung tissue of subjects with COPD [8].

Numerous studies in the last decade have shown distinct macrophage subpopulations, each with its own distinct functional properties [9]. Traditionally macrophages are phenotyped as either “classically” activated or M1 macrophages, or “alternatively” activated or M2 macrophages, where M1 macrophages are more pro-inflammatory and have cytotoxic properties to process foreign particulate matter, including pathogens, while M2 macrophages are anti-inflammatory and are involved in inflammation resolution and tissue repair processes [1,9]. Several subpopulations of these two basic phenotypes have been described, for example, M2a, M2b and M2c, each with their own distinct physical and functional properties [10]. The diversity and plasticity of lung macrophages have been well documented using in vitro transformed monocytes or monocyte cell lines and animal models. Whether there are additional distinct phenotypes and whether the distribution of these phenotypes changes with diseases such as COPD, in which macrophages are thought to have a key role in the pathogenesis of the disease [11,12,13,14,15], is still unclear.

Bazzan and co-workers described the presence of non-polarized macrophages (i.e., macrophages that are negative for both traditional M1 and M2 markers) [11], and Eapen and co-workers reported on dual-polarized macrophages, which express both M1 and M2 markers [12]. Preliminary studies from our laboratory confirmed the presence of these sub-populations of alveolar macrophages [16], but little is known about their functional properties or gene expression profiles in COPD [16,17]. The goal of this study was to quantify the distribution of these different macrophage subpopulations (M1, M2, double-polarized, and non-polarized macrophage) across the severity of COPD using macrophages harvested from broncho-alveolar lavage (BAL) fluid and characterize their functional properties in COPD patients.

## 2. Methods

### 2.1. Subjects

We recruited stable COPD patients who had been free of exacerbations for at least 4 weeks prior to sample collection, and a control group, which consisted of individuals who did not have COPD but underwent bronchoscopies for a clinical indication (i.e., for lung nodules or masses, chronic cough, or mediastinal lymphadenopathy) between 2017 and 2019 at St. Paul’s Hospital in Vancouver, Canada (ClinicalTrials.gov identifier: NCT02833480, 19/02/2015). All subjects provided written informed consent and the research protocol was approved by the University of British Columbia/Providence Health Care Research Ethics Committee (certificate numbers: H14-02277, 19/02/2015). The diagnosis of COPD and severity score were based on the Global Initiative for Chronic Obstructive Lung Disease (GOLD) criteria [18].

### 2.2. Broncho-Alveolar Lavage (BAL) Collection

Using a fiberoptic Olympus^®^ bronchoscope (Olympus Corporation, Tokyo, Japan), the patients’ 4–6th generation airways were cannulated and then wedged. The target segment(s) had to be free of any significant disease based on chest computed tomography (CT) imaging and not contralateral to any significant lung pathology including pulmonary nodules or mass, or bronchiectasis. BAL was performed by instilling 40 mL of sterile normal saline into an occluded segment with subsequent 20 mL aliquots (to a maximum of 200 mL) until a total of 50 mL of BAL fluid was retrieved. The first 20 mL of the collection fluid was discarded.

### 2.3. Alveolar Macrophages

Macrophages were purified on the day of collection from the BAL fluid as previously described in detail [19]. Briefly, BAL fluid was filtered through Cell strainer 70 μm pore size (VWR, Randor, PA, USA) and centrifuged at 500 relative centrifugal force (rcf) for 10 min, at 4 °C. The total cell number was counted with a hemocytometer. BAL samples were processed under sterile conditions within 1 h after collection and maintained on ice until processed.

### 2.4. Inmunofluorescence Staining and Flow Cytometric Analysis

We used 10% human serum for 20 min at 4 °C to block nonspecific Ab binding. The cells were stained with anti-human HLA-DR antibody (APC/Cy7, clone L243, Biolegend, San Diego, CA, USA) (to identify macrophages), anti-human CD40 antibody (Brilliant Violet 421TM, clone 5C3, Biolegend, San Diego, CA, USA) to identify M1 polarization, anti-human CD163 antibody (Alexa Fluor 647, clone GHI/61, Biolegend, San Diego, CA, USA) to identify M2 polarization. Appropriate isotype controls were used for each antibody. These M1 (CD40) and M2 (CD163) markers gave use the clearest and strongest signals and were selected after testing a variety of other different M1 (CD80, CD86, iNOS) and M2 (CD206) markers (Figure S1). We incubated the cells and the antibodies for 60 min on ice in the dark, and after washing the cells, we evaluated them using a Gallios flow cytometer and cell sorter MoFlo Astrios EQ (Beckman Coulter Life Sciences, Indianapolis, IN, USA). The data were analyzed using Kaluza Analysis Software (Beckman Coulter). Macrophages were gated into four categories: M1 [CD40+, CD163−, HLA-DR+], M2 [CD40−, CD163+, HLA-DR+], double-polarized or Double Positive (DP) [CD40+, CD163+, HLA-DR+], and non-polarized or Double Negative (DN) [CD40−, CD163−, HLA-DR+]). An example of the gating strategy is shown in Figure S1.

### 2.5. Measurement of Macrophage Phagocytosis

We used pHrodo^TM^ Red *S. aureus* bioparticle conjugates^TM^ (Invitrogen, Carlsbad, CA, USA) in order to evaluate the phagocytic activity of macrophages [20]. The cells were washed in phosphate-buffered saline (PBS). Macrophages (1 × 10^6^/mL) were incubated with the pHrodo-labeled bioparticles in a 37 °C water bath for 2 h and then collected for flow cytometry analysis according to the manufacturer’s recommendations. This assay is based on the principle that a fluorescence signal dramatically increases in response to the lower pH of the phago-lysosome, which occurs with macrophage engulfment of the bioparticles [20]. The fraction of pHrodo-positive cells (indicating phagocytic activity) was determined for each macrophage phenotype.

### 2.6. Gene Expression Measurement/Analysis

After macrophages were divided into four groups, they were transferred to an RLT buffer (Qiagen, Hilden, Germany) and stored at −80 °C. The nCounter^®^ GX Human Inflammation Kits (Nanostring Technologies, Seattle, WA, USA) were used to analyze gene expression. We had 48 RNA samples for this part of the experiment (seven non-COPD and five COPD subjects). The panel profiles included 255 targeted genes (249 inflammation-related genes and 6 internal reference controls). Total RNA (extract from > 5000 cells) was assayed on a nCounter Digital Analyzer (NanoString Technologies, Seattle, WA, USA) according to the manufacturer’s protocol. Gene expression was analyzed on the accompanying nSolver software (NanoString Technologies, Seattle, WA, USA). Raw count data of gene expression was processed according to NanoString’s recommendations. Data were normalized to the average of the 6 housekeeping genes (CLTC, GAPDH, GUSB, HPRT1, PGK1, and TUBB) in each experimental sample and log2-transformed for further analysis for differential expression (DE).

### 2.7. Statistical Analysis

The statistical software PRISM 5 (GraphPad Software, Inc., San Diego, CA, USA) was used for Fisher’s exact test for tables (2 × 2), the Mann-Whitney U test, Kruskal–Wallis test and post hoc Dunn’s test with Bonferroni adjustment for multiple comparisons, and Spearman rank correlation test, as appropriate. *p* < 0.05 was considered significant.

### 2.8. Gene Expression Analysis

For differential gene expression analysis, the raw count data were quality controlled and normalized using the positive control genes and housekeeping genes, according to NanoString’s recommendation. Principal component analysis was used to assess batch effect and outliers (Figure S2). Since we had observed a batch effect between experiments, normalization was performed on each experiment separately and then combined using ComBat (R package “sva”) batch correction. The processed data were log2-transformed, and genes with log2 expression < 4 in at least 12 samples (1/4 of the total sample size) were filtered out prior to the downstream analysis.

Following the classical definition of cell markers, differential expression analysis was performed using R package limma’s moderated linear model comparing one cell type versus the others. To account for the intra-subject correlation between samples, we used the mixed effect version of the model. The Benjamini-Hochberg procedure was used to correct for multiple hypothesis testing and a false discovery rate < 0.1 was used as the significance threshold. Linear regression was used to examine the association between phenotypes (i.e., sex, age, smoking status and disease status) and the first five principal components of the expression data to determine the potential covariates. R package “clusterProfiler” was used for pathway enrichment analysis. All analyses were performed using R (version 3.5.0).

## 3. Results

### 3.1. Patient Characteristics

The characteristics of 28 study subjects (18 COPD and 10 control) are shown in Table 1.

In the COPD group, there was more males (78% vs. 30%, *p* = 0.020), a higher pack-year smoking history (median 39.3 [IQR 19.3–48.8] vs. 0.0 [0.0–1.5], *p* < 0.001), and lower forced expiratory volume in one second (FEV_1_) % predicted (68.5% [53.2–73.8%] vs. 95.0% [81.6–104.2%], *p* = 0.001) and FEV_1_/forced vital capacity (FVC) ratio (60.8% [50.9–68.4%] vs. 77.8% [74.6–82.3%], *p* < 0.001) than controls. The GOLD stages I/II/III/VI were 4/10/4/0. Both groups were similar in terms of age and percentage of FVC % predicted.

### 3.2. Macrophage Phenotype Distribution

**Figure 1:**
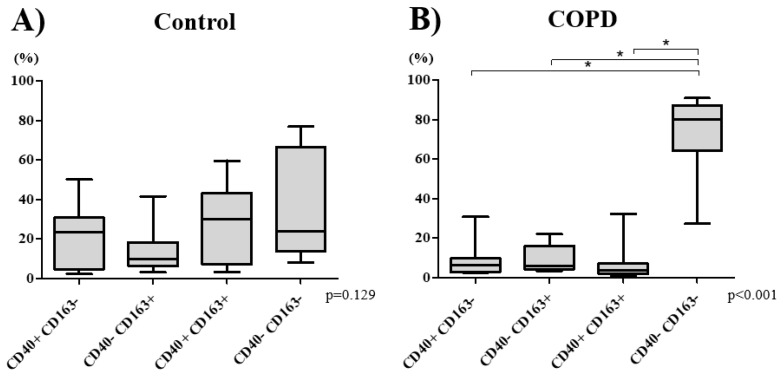
The percentage of macrophage subtype was compared between control (**A**) and COPD (**B**) subjects. There was no significant difference in the percentage of macrophage subtypes in control subjects (**A**). The percentage of non-polarized (CD40-CD163-) macrophage was the highest in COPD subjects (**B**). Bottom and top of each box represent 25th and 75th percentiles, respectively; the solid line indicates median; brackets represent 10th and 90th percentiles. *p* values were determined using a Kruskal–Wallis test and a post hoc Dunn’s test with Bonferroni adjustment for multiple comparisons. * *p* < 0.05. Definition of abbreviations: COPD chronic obstructive pulmonary disease.

In control subjects, there was a near-equal distribution of the four subtypes of macrophages (*p* = 0.129): M1^CD40+CD163−^ (Median 22.9% [IQR 8.4–28.0%]), M2^CD40−CD163+^ (9.5% [6.5–16.0%]), double-polarized^CD40+CD163+^ (28.9% [11.1–38.8%]), and non-polarized macrophages^CD40−CD163−^ (23.9% [14.6–51.5%]) (Figure 1A). In the COPD group, however, there was a significant increase in the percentage of non-polarized macrophages (77.9% [62.6–83.8%]) compared with those of other macrophage subtypes M1 5.8% [2.9–9.2%], M2 6.0% [4.0–14.4%], and double-polarized 4.3% [2.2–6.6%], respectively (*p* < 0.001) (Figure 1B). COPD patients had significantly lower percentage of both double-polarized and a higher percentage of non-polarized macrophages that those in the control group (*p* < 0.001, Table S1).

### 3.3. COPD Severity and Distribution of Macrophage Subtypes

**Figure 2:**
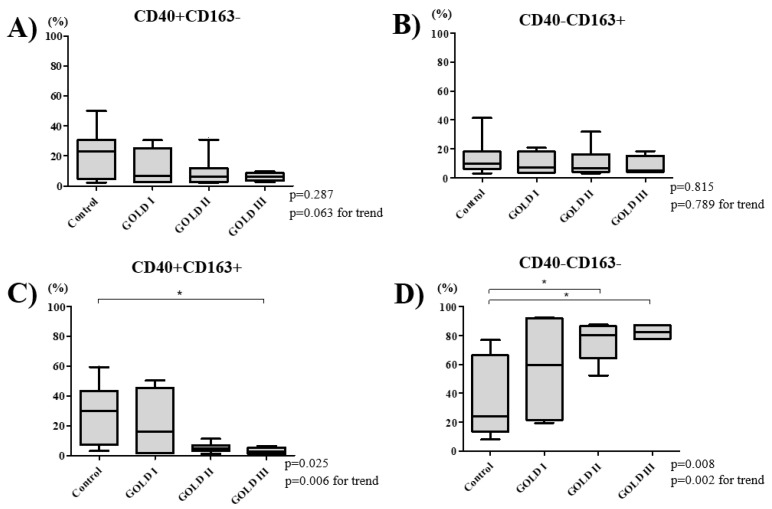
Percentage of M1 (CD40+CD163-) (**A**), M2 (CD40-CD163+) (**B**), double-polarized (CD40+CD163+) (**C**), and nonpolarized (CD40-CD163-) macrophages (**D**) was compared between control and COPD subjects across GOLD grades of severity. The percentage of M1, M2, and double-polarized macrophages macrophage decreased (A, B, and C), while the percentage of non-polarized macrophages increased with disease progression (**D**). *p* values were determined using a Kruskal–Wallis test and a post hoc Dunn’s test with Bonferroni adjustment for multiple comparisons. *p* value for trend was calculated to determine the significance across the GOLD grades of severity. * *p* < 0.05. Definition of abbreviations: COPD chronic obstructive pulmonary disease, GOLD Global Initiative for Chronic Obstructive Lung Disease.

**Figure 3:**
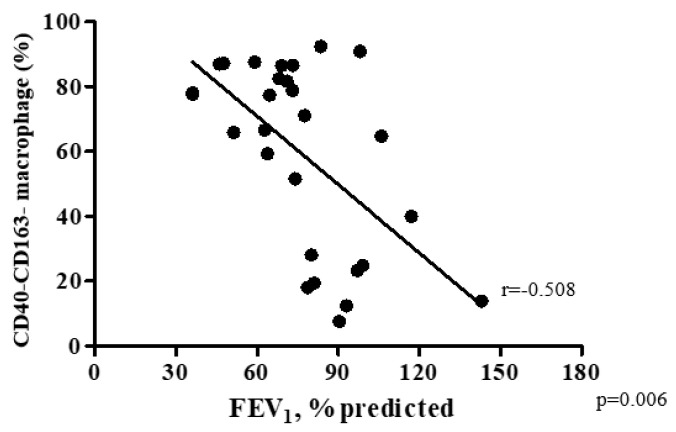
Percentage of non-polarized (CD40-CD163-) macrophages was negatively correlated with FEV1 (%predicted). Spearman rank correlation r = −0.508 and *p* = 0.006. Definition of abbreviations: FEV1 forced expiratory volume in one second.

**Figure 4:**
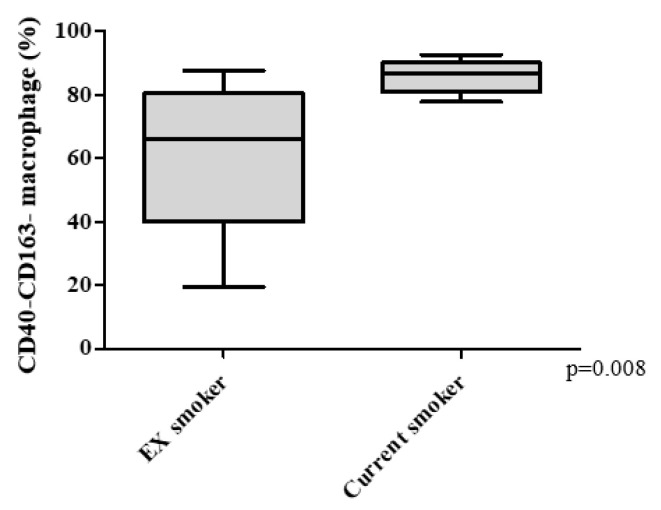
The percentage of non-polarized (CD40-CD163-) macrophages in current smokers was significantly higher than in that in ex-smokers among COPD subjects. *p* value was determined using a Mann-Whitney U test. Definition of abbreviations: COPD chronic obstructive pulmonary disease.

The percentage of double-polarized macrophages decreased and non-polarized macrophages increased with increasing COPD severity (Figure 2C,D). The percentage of M1 macrophages was 6.5% [3.5–14.5%] in GOLD 1, 5.8% [2.9–8.7%] in GOLD II, and 5.8% [4.6–7.0%] in GOLD III grade of severity (*p* = 0.063; Figure 2A) and the percentage of M2 macrophage was 7.4% [1.5–13.5%] in GOLD 1, 6.6% [4.1–14.4%] in GOLD II, and 5.3% [4.1–8.6%] in GOLD III (*p* = 0.789; Figure 2B). The percentage of the double-polarized macrophages was highest in GOLD 1 at 15.8% [1.5–35.0%], which decreased to 4.7% [3.2–6.3%] in GOLD II, and 2.4% [1.7–3.6%] in GOLD III (*p* = 0.025; Figure 2C). Conversely, the percentage of non-polarized macrophages increased from 57.2% [25.8–87.3%] in GOLD 1, to 77.6% [62.9–79.9%] in GOLD II and 78.9% [74.2–81.6%] in GOLD III (*p* = 0.008; Figure 2D). There was a significant inverse relationship between FEV1% predicted and the percentage of non-polarized macrophage in BAL fluid (Spearman rank correlation r = −0.508 and *p* = 0.006; Figure 3). The percentage of non-polarized macrophages was also higher in current smokers compared with ex-smokers (Figure 4).

### 3.4. Phagocytosis Macrophage Subtypes

**Figure 5:**
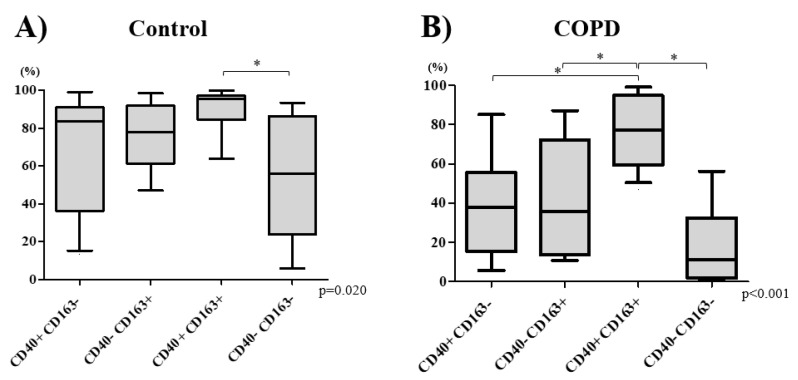
The percentage of pH-rodo Red *Stapylococcus aureus* bioparticles conjugate posimacrophages (phagocytic activity) in each macrophage subtype was compared between control (**A**) and COPD (**B**) subjects. The phagocytic activity of non-polarized (CD40-CD163-) macrophage was lower than double-polarized (CD40+CD163+) macrophages in control subjects (**A**). The phagocytic activity of double-polarized (CD40+CD163+) macrophage was the highest in COPD subjects (**B**). Bottom and top of each box plot represents 25th and 75th percentiles, respectively; the solid line indicates the median; and the brackets denote 10th and 90th percentiles. *p* values were determined using a Kruskal–Wallis test and a post hoc Dunn’s test with Bonferroni adjustment for multiple comparisons. * *p* < 0.05.

Among COPD patients, the double-polarized macrophages demonstrated the highest phagocytic activity (Median 77.4% [IQR 62.8–93.3%]). Next was M1 macrophages (38.0% [16.8–52.8%]), which was followed by M2 macrophages (35.7% [14.0–68.7%]), with worst phagocytic index in the non-polarized macrophages (11.0% [2.4–31.0%]; *p* < 0.001; Figure 5B). In control subjects, the phagocytic activity of non-polarized macrophages (56.1%, [30.7–80.6%]) was also significantly lower than that of double-polarized macrophages (95.7% [89.5–96.2%]; *p* < 0.05) (Figure 5A).

**Figure 6:**
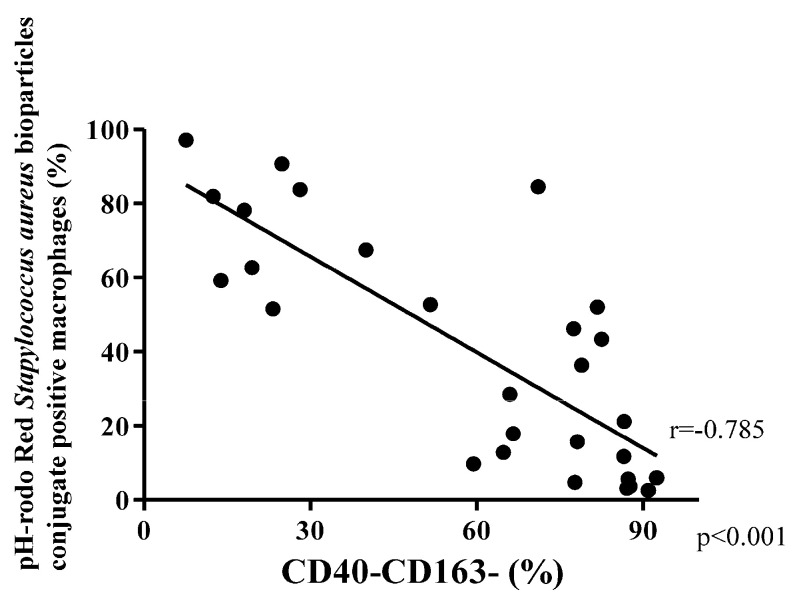
The percentage of non-polarized (CD40-CD163-) macrophages was negatively correlated with the phagocytic activity of macrophages (percentage of pH-rodo Red *Stapylococcus aureus* bioparticles conjugate positive macrophages). Spearman rank correlation *r* = −0.785 and *p* < 0.001.

The phagocytic activity of M1, M2, and non-polarized macrophages in COPD subjects in general were lower than those in control subjects (*p* = 0.020, *p* = 0.006, and *p* = 0.004, respectively) (Table S2). There was a trend towards reduced phagocytic activity of double-polarized macrophages in COPD subjects compared to control subjects (*p* = 0.068; Table S2). There was an inverse relationship between the percentage of non-polarized macrophages in BAL fluid and phagocytic activity in all participants (Spearman rank correlation r = −0.785 and *p* < 0.001; Figure 6).

### 3.5. Gene Expression in Macrophages

**Figure 7:**
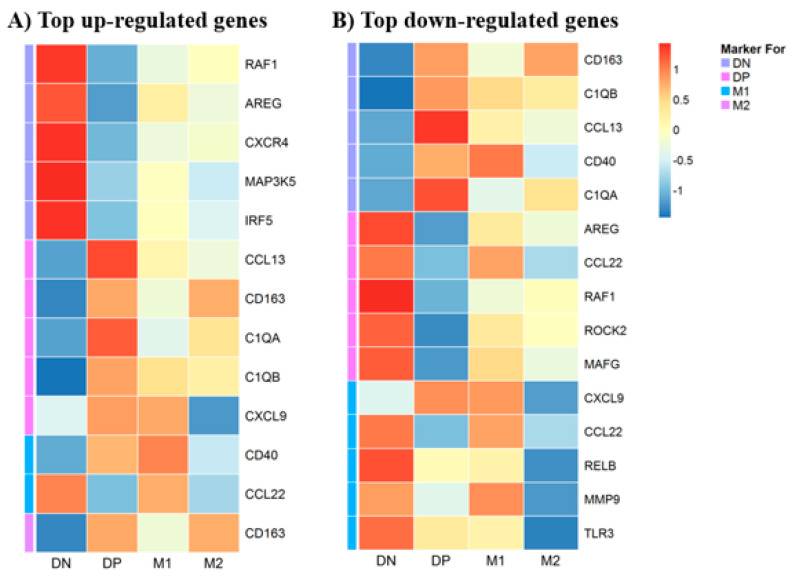
Hear map showing the mean scaled expression of the differentially expressed genes in each macrophage subtype compares to other macrophages subtypes (all study subjects) ((**A**): Top up-regulated genes, (**B**): Top down-regulated genes).

**Figure 8:**
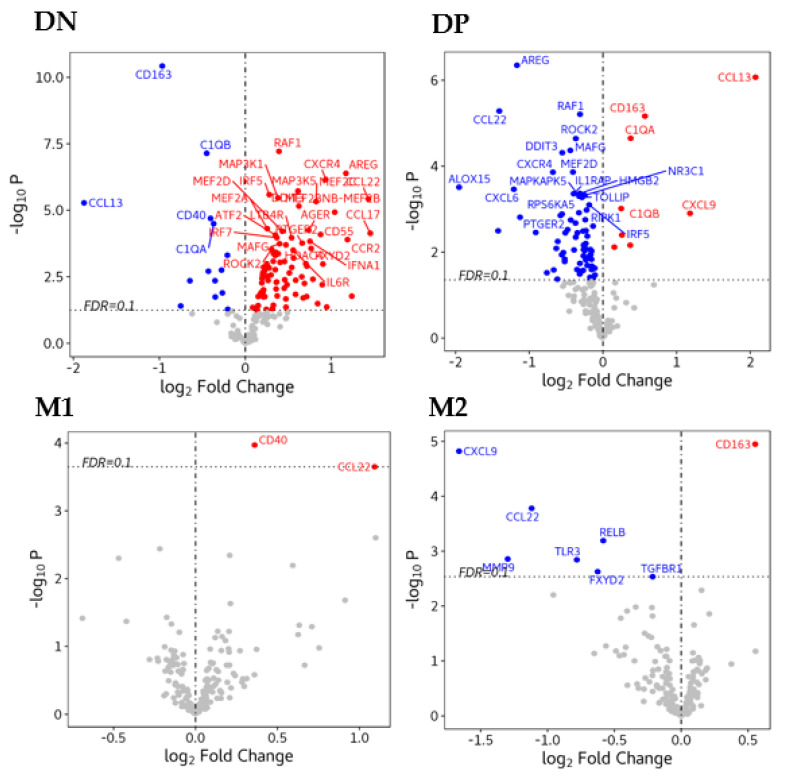
Volcano plots show the gene expression graphically DN (top left, double negative), DP (top right), M1 (bottom left, double positive), M2 (bottom right). Blue = down-regulated in each subtype, red=up-regulated in each subtype, top genes were labeled with their symbols.

We used a multiplex gene expression panel, consisting of 249 relevant genes, which are known to be involved in the initiation, propagation and resolution of inflammation to evaluate the molecular profile of BAL macrophage subtypes. By checking the association between the variables (age, sex, disease conditions and smoking status) and the first five principal components, no significant covariates were included in the differential expression analysis (all *p* > 0.05). The differential gene expression analysis showed that 90 genes were up-regulated and 14 genes were down-regulated in non-polarized macrophages (Figure S3A,B); whereas in double polarized macrophages, 8 genes were up-regulated and 73 genes were down-regulated (Figure S3C,D). In contrast, only 2 genes were up-regulated and no genes were down-regulated in M1 macrophages (Figure S3E) and only 1 gene was up-regulated and 7 genes were down-regulated in M2 macrophages (Figure S3F,G). Heat maps of the top up-regulated and top down-regulated DE genes in each macrophage phenotype are shown in Figure 7A and Figure 7B respectively. Volcano plots graphically show gene expression in the four different macrophage populations (Figure 8). No enriched Gene Ontology and KEGG pathways were identified at false discovery rate < 0.1.

## 4. Discussion

Macrophages play a pivotal role in the chronic inflammatory response in COPD lungs. Here we showed that among COPD patients, the majority of macrophages in BAL fluid are non-polarized; whereas in non-COPD subjects, these macrophages constitute ~25% of the total pool of macrophages. Several studies have shown an increase in the number of macrophages in bronchoalveolar lavage fluid in smokers and COPD subjects compared with non-smoking controls [4,5]. We also showed that the proportion of these non-polarized macrophages increases with increasing COPD severity, suggesting that these macrophages could contribute to the progression of disease, though additional studies will be required to establish causality. Previous studies have shown that lung macrophages in COPD have reduced phagocytic ability [17,21,22], and here we showed that these non-polarized macrophages predominantly contribute to this impaired phagocytic activity. This reduced phagocytic activity of non-polarized macrophages was also related to the severity of the underlying COPD. Together, these findings support the notion that these non-polarized macrophages contribute to the inflammatory milieu in the lung tissues and also could contribute to enhanced risk of exacerbations and progression of COPD.

Although we found increased abundance of non-polarized macrophages in COPD subjects compared to controls, there were no fractional differences in the four different macrophage subtypes in the control group. We also found that that the percentage of double-polarized macrophages was significantly lower in the COPD patients compared with that in control subjects (*p* = 0.007; Table S1). The clinical relevance of this observation, however, is unclear, although this phenotypic shift of macrophages in COPD supports the notion that these non-polarized macrophages contribute to the persistent inflammatory responses in lung tissues of COPD [6,7]. A few studies have previously addressed the issue of macrophage phenotypes shifts in COPD [11,12,13,14,15,16,17]. Kunz and coworkers, for example, used induced sputum samples (which represents mostly macrophages from the larger airways) to evaluate M1 and M2 phenotypes [15], while Eapen and co-workers [12], using macrophages harvested from BAL fluids, evaluated different phenotypes of macrophages and found that the percentage of non-polarized macrophage increased in COPD subjects; results that were similar to the findings of the present study. We extend these findings by showing a severity-dependent relationship between COPD GOLD grades and the percentage of non-polarized macrophages in BAL fluid of COPD subjects. The M1 and M2 macrophage distribution in control subjects (Figure 1A) is similar than those reported by Eapen [12] and Shaykhiev [14]. However, it should be noted that there are slight differences in the percentages of M1 and M2 macrophages that have been reported in the literature, which may be related to differences in the cell surface markers employed by each of the studies to identify these subtypes. For example, Hodge and coworkers used MHC class I and class II to capture M1 macrophages [13], whereas Löfdahl [23] and coworkers used CR-3 as the cell surface marker for M1 macrophages.

Here, we also showed that current smokers have increased percentage of non-polarized macrophages in BAL (Figure 4). Taken together with previous studies, our current findings support the concept that lung macrophages change their phenotype depending on disease status and environmental stimuli (such as smoking). We suspect that this macrophage plasticity is predominantly influenced by the local microenvironment [24]. The chronic and persistent inflammatory milieu in COPD lung tissue (or induced by inhalation of CS or PM) may recruit monocytes from the peripheral circulation into the lung tissue and/or airspaces [8,25] where they differentiate and become macrophages; these monocyte-derived macrophages may be initially non-polarized [17]. One potential source of non-polarized macrophages could be these “newly” recruited cells. Alternatively, these non-polarized macrophages could be macrophages that have lost their M1 or M2 characteristics (markers) over time and are destined for apoptosis and removal [17,25]. Leukocyte kinetic studies are necessary to address this issue.

Our study showed that non-polarized macrophages have poor receptor-mediated phagocytic capacity, which was observed in both control and COPD subjects (Figure 5; Figure 6). Previous studies using a mixture of all lung macrophages in COPD subjects and in cigarette smoke-treated animals, have shown decreased phagocytic function of macrophages in response to microorganisms such as *Streptococcus pneumoniae*, *Haemophilus influenzae*, *Escherichia coli*, *Moraxella catarrhalis*, and *Candida albicans* [21,22,26,27,28,29,30,31]. To our knowledge, our study is the first to explore phagocytic function of distinct populations of broncho-alveolar macrophages in humans. We suspect that the low phagocytic function in these previous reports is due to the abundance of non-polarized macrophages in these samples. In contrast, we showed that the double positive macrophages demonstrated excellent phagocytic function (Figure 5). We posit that the increased presence of non-polarized macrophages (at the expense of double positive macrophages) in COPD airways may increase the risk of these patients risk to repeated infections and exacerbations [32].

Previous studies have shown differences in gene expression between M1 and M2 macrophages derived from human peripheral blood mononuclear cells (PBMC) [33,34] or cultured human monocytes [35,36]. Differential gene expression of subtypes or phenotypes of macrophages retrieved from the airspaces or lung tissues of subjects with COPD is still unclear. Using heat maps of differentially expressed genes, we also showed that CD40 was up-regulated in double-polarized and M1 macrophages and down-regulated in non-polarized macrophages while CD163 was up-regulated in double-polarized and M2 macrophages and down-regulated in non-polarized macrophages (Figure S3 and Figure 7A,B). These findings support our flow cytometric data. CD40 is a costimulatory receptor related to antigen presenting cells [37] and CD163 is a scavenger receptor [38,39] related to phagocytosis of a variety of particulate matter. The phagocytic function of polarized M1, M2 or double positive macrophage was higher than that of non-polarized macrophages, suggesting that these two receptors CD40 and CD163 contribute to these macrophages’ ability to recognize and process foreign materials including pathogens. In the non-polarized macrophages, 14 genes were down-regulated, including CD40 and CD163, which may impair their capacity for pathogen recognition and processing. In addition, the complement components C1QA and C1QB were also down-regulated in non-polarized macrophages (Figure 8). C1Q is a pattern recognition protein that binds to antibody–antigen complexes, bacteria, and viruses and stimulates phagocytic function of human monocytes and macrophages [40,41]. Furthermore, C1Q also directs macrophage polarization and limits inflammasome activity during the uptake of apoptotic cells [42] and promotes M2 polarization [43], both key functions to assist in inflammation resolution and tissue repair. Attenuation of these key macrophage functions could promote bacterial colonization and subsequent infectious exacerbations in COPD and also contribute to dysregulated resolution of the downstream inflammatory responses and impaired tissue repair.

The *RAF1* is part of a signaling pathway called the RAS/MAPK pathway, which transmits signals from outside the cell to the cell’s nucleus to promote cell division (proliferation), cell maturation and differentiation, cell recruitment and eventually apoptosis [44,45]. AREG or amphiregulin is an Epidermal Growth Factor (EGF)-like molecule that has recently been shown to play a central role in orchestrating both host resistance to pathogens and immune tolerance mechanisms [46,47,48]. This cross-talk between immune cells (such as macrophages) and epithelial cells are programmed to promote inflammation resolution and tissue regeneration, leading to homeostasis after injury [49]. However, with a chronic persistent inflammatory stimulus such as cigarette smoking, it could promote proliferation of structural cells such as fibroblasts and their production of pro-inflammatory cytokines such as IL-8, vascular endothelial growth factor (VEGF), and transforming growth factor alpha (TGF-α) with augmented expression in chronic inflammatory conditions such as rheumatoid arthritis [50]. Mitogen-activated protein kinase 5 (MAP3K5) is a member of MAP kinase family that activates c-Jun N-terminal kinase (JNK) and p38 mitogen-activated protein kinases to an array of stresses such as oxidative stress, endoplasmic reticulum stress and calcium influx [51,52,53]. It has been implicated in the pathogenesis of chronic inflammatory conditions such as rheumatoid arthritis, cardiopulmonary diseases and diabetes [54,55]. Lastly, CXCR4 is a chemokine receptor for pro-inflammatory mediators such as macrophage migration inhibitor factor (MIF), which is a pleiotropic cytokine that antagonizes both apoptosis and premature senescence [56,57,58]. It has been shown to be elevated and implicated in the pathogenesis of COPD [59]. Together, upregulation of these genes suggest that these non-polarized macrophages were also pro-inflammatory in nature and suppress inflammation resolution and tissue repair. Therefore, this abundance of non-polarized macrophages could significantly contribute to the chronic persistent lung inflammatory response in COPD. Taken together, the gene expression profile of these non-polarized macrophages suggests a perturbed pathogen recognition and processing function and reduced ability to resolve inflammation and promote tissue repair (Figure S3, Figure 7A,B and Figure 8).

There were limitations in this study. Firstly, we did not have a group of smokers with normal lung function to determine the independent effects of cigarette smoking on macrophage phenotypes. However, we found that the percentage of non-polarized macrophages in current smokers with COPD was higher than that of ex-smokers with COPD, suggesting that there is likely to be an effect of current smoking on macrophage plasticity. Given the relatively small number of current smokers in the COPD group and that there were no current smokers in the control group, the independent effect of active cigarette smoking on lung macrophage phenotype and function needs to be explored in a larger dedicated study. Secondly, we performed targeted profiling of 249 human genes known to be involved in different pathways of inflammation rather than unbiased sequencing. Thus, we may have missed other important processes or pathways related to macrophage subtypes in both health and disease. A priori, we chose NanoString targeted gene array, owing to its high sensitivity, specificity and accuracy for the targeted inflammatory genes. Lastly, the origin of these non-polarized macrophages is still unclear. We postulate two possible pathways, firstly, that they represent newly recruited blood monocytes (in contrast to resident macrophages) that have not fully mature with their full complement of functions including their phagocytic function or, alternatively, that these dual negative macrophages are senescent macrophages that have lost both their M1 and M2 markers, including their phagocytic abilities and are destined for removal by apoptosis. Due to the lack of specific markers that can identify recently recruited macrophages in lung tissues, monocyte kinetic studies [60,61] are needed to clarify this important issue. Classification of macrophages in general has been proposed [62]; however, whether this will be applicable to a unique population such as alveolar macrophages still requires clarification. Single cell genetic analysis may provide useful information in future studies not just to better classify lung macrophages phenotypes [63], but also to give insight into the functional properties of these subpopulations of macrophages.

## 5. Conclusions

In this study, we identified a unique population of macrophages in human BAL samples of subjects with COPD that lack two important M1 (CD40) and M2 (CD163) markers and are poorly or non-polarized. These non-polarized macrophages constitute the majority of macrophages in COPD, and their abundance increases with increasing COPD severity. These non-polarized macrophages are also pro-inflammatory in nature, with poor phagocytic function. The reduced capability of these non-polarized macrophages to phagocytose bacteria could increase vulnerability for COPD exacerbations. Further studies are needed to determine whether these non-polarized macrophages can be therapeutically targeted, either to change to functional M1 or M2 macrophages or be removed. Therapeutically targeting these macrophages may reduce COPD exacerbations and slow the progression of COPD.

## Figures and Tables

**Table 1:** Characteristics of control and COPD patients.

Characteristics	Control (*n* = 10)	COPD (*n* = 18)	*p* Value
Age, years	65.0 (60.8–68.8)	65.0 (60.5–70.8)	0.665
Sex, male	3 (30)	14 (78)	0.020
Smoking status			
Current	0 (0)	8 (44)	0.025
Ex	3 (30)	9 (50)	0.434
Never	7 (70)	1 (6)	<0.001
Pack-year smoked	0.0 (0.0–1.5)	39.3 (19.3–48.8)	<0.001
Pulmonary function			
FVC, % predicted	94.6 (85.5–109.3)	85.0 (72.8–95.7)	0.144
FEV_1_, % predicted	95.0 (81.6–104.2)	68.5 (53.2–73.8)	0.001
FEV_1_/FVC	77.8 (74.9–82.3)	60.8 (50.9–68.4)	<0.001
GOLD stage I/II/III/IV	-	4/10/4/0	
BAL findings			
Total cell count (X10^5^/mL)	0.8 (0.3–1.3)	1.3 (0.5–2.3)	0.352
Macrophages (%)	96.0 (95.0–96.5)	94.0 (92.0–97.0)	0.445
Lymphocytes (%)	1.5 (0.8–2.3)	2.0 (1.0–5.0)	0.369
Neutrophils (%)	2.0 (1.0–2.3)	1.0 (1.0–3.0)	1.000
Eosinophils (%)	0.0 (0.0–1.0)	0.0 (0.0–0.8)	0.646
Inhalation therapy			
Formoterol (%)	0 (0)	1 (5.6)	0.643
Budesonide/Formoterol (%)	0 (0)	2 (11.1)	0.405
Fluticasone/Salmeterol (%)	0 (0)	4 (22.2)	0.149

Data are presented as median (interquartile range). Definition of abbreviations: COPD chronic obstructive pulmonary disease, FVC forced vital capacity, FEV1 forced expiratory volume in one second, GOLD Global Initiative for Chronic Obstructive Lung Disease. BAL bronchoalveolar lavage.

## Supplementary Materials

The following are available online at https://www.mdpi.com/2227-9059/8/10/398/s1, Figure S1: Flow cytometry gating strategy; Figure S2: PCA plots and gate corrections; Figure S3: Gene expression in different macrophage phenotypes; Table S1: Percentage of macrophage subtypes in control and COPD subjects: Table S2: Macrophage phagocytosis of Staphylococcus Aureus in control and COPD subjects.

## Data and Material Availability

The datasets used and/or analyzed during the current study are available from the corresponding author on reasonable request.
