# Supplementary material for: Abundance of Non-Polarized Lung Macrophages with Poor Phagocytic Function in Chronic Obstructive Pulmonary Disease (COPD)
Source: Biomedicines. 2020 Oct 8;8(10):398. doi: 10.3390/biomedicines8100398 (PMC7650830; doi:10.3390/biomedicines8100398)
Supplement: Supplementary file 1 [file biomedicines-08-00398-s001.pptx]

## Slide 1
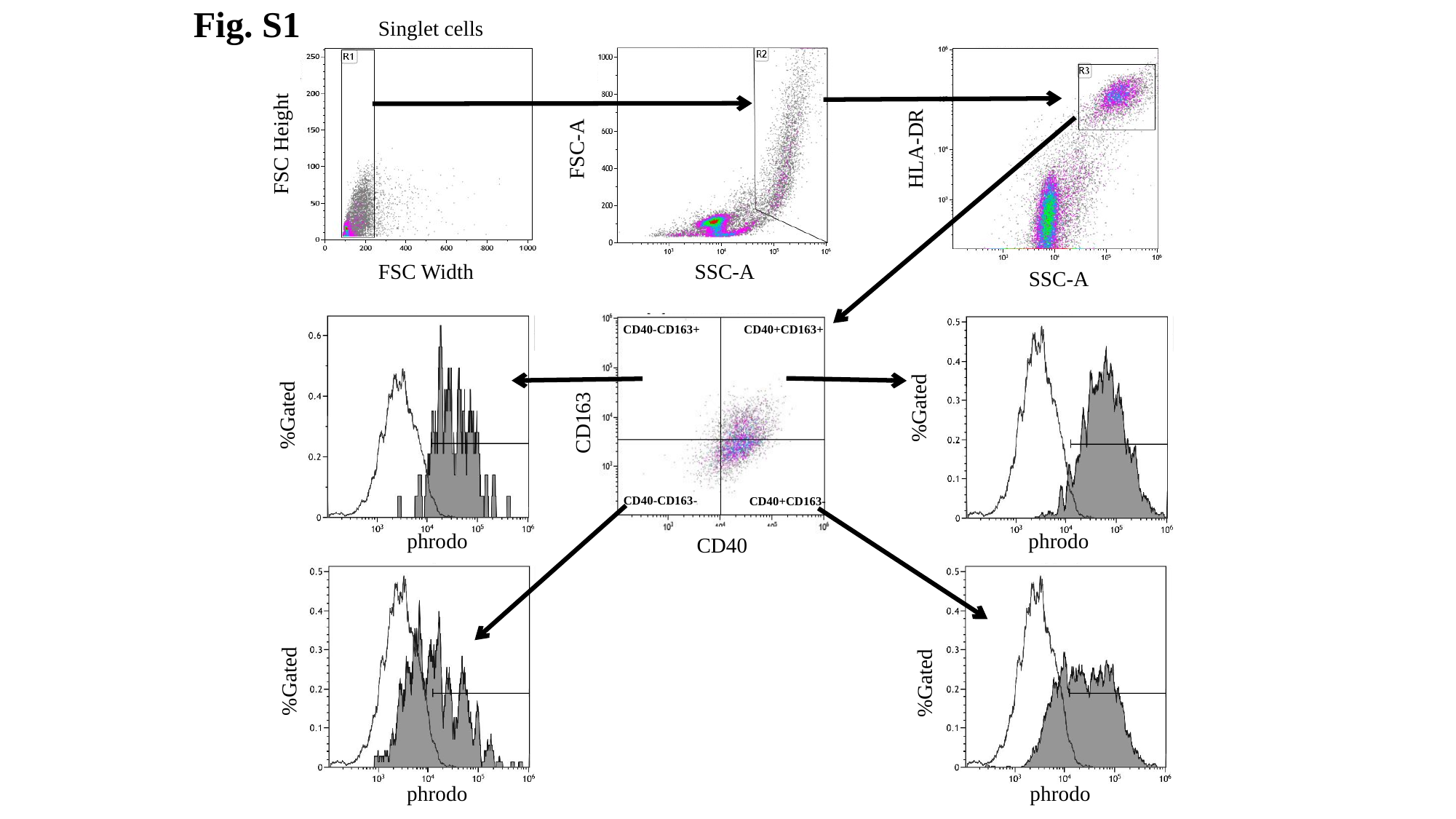

Fig. S1
Singlet cells
FSC Height
FSC-A
HLA-DR
FSC Width
SSC-A
SSC-A
CD40-CD163+
CD40+CD163+
%Gated
%Gated
CD163
CD40-CD163-
CD40+CD163-
phrodo
phrodo
CD40
%Gated
%Gated
phrodo
phrodo

## Slide 2
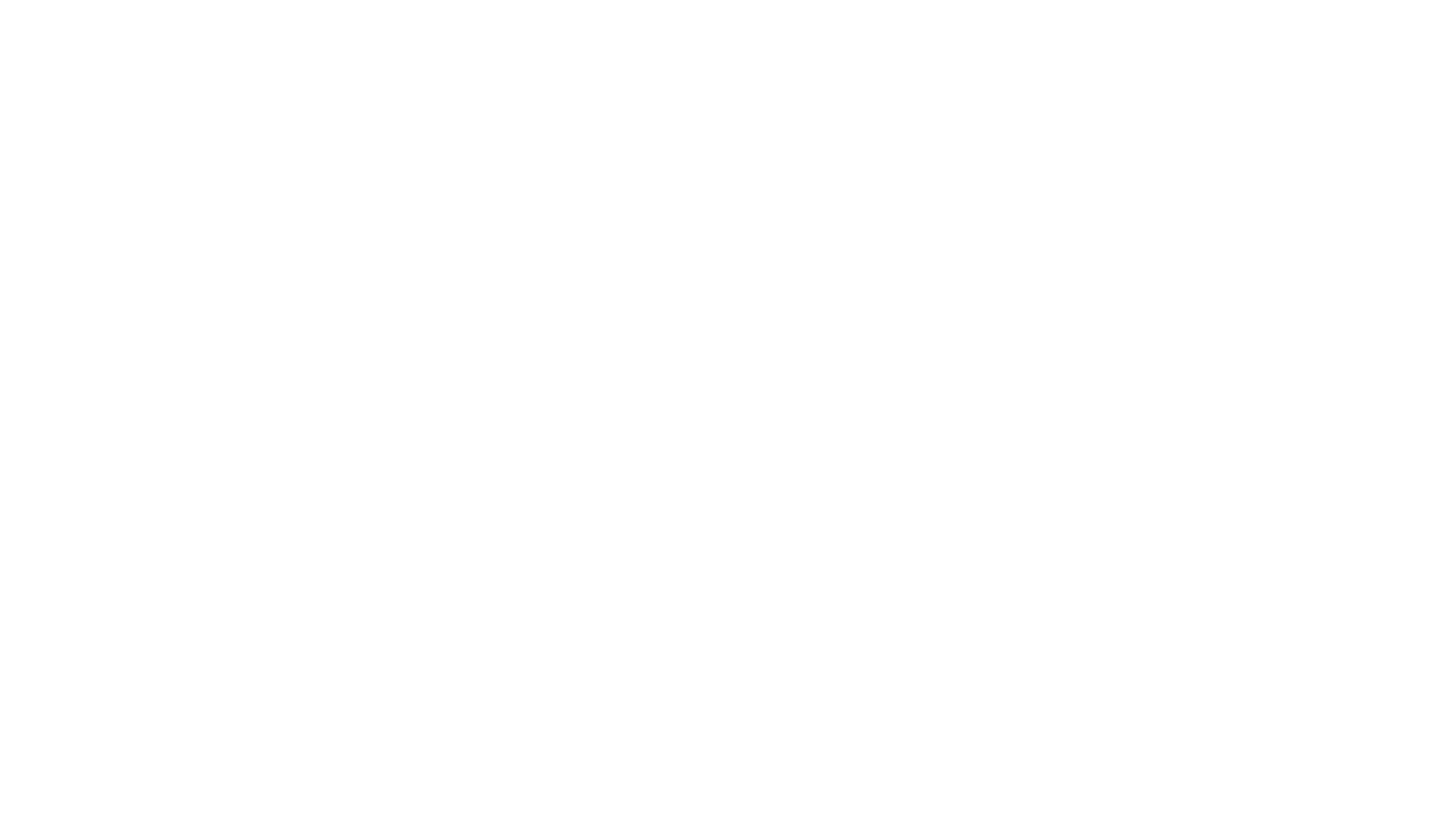

#

## Slide 3
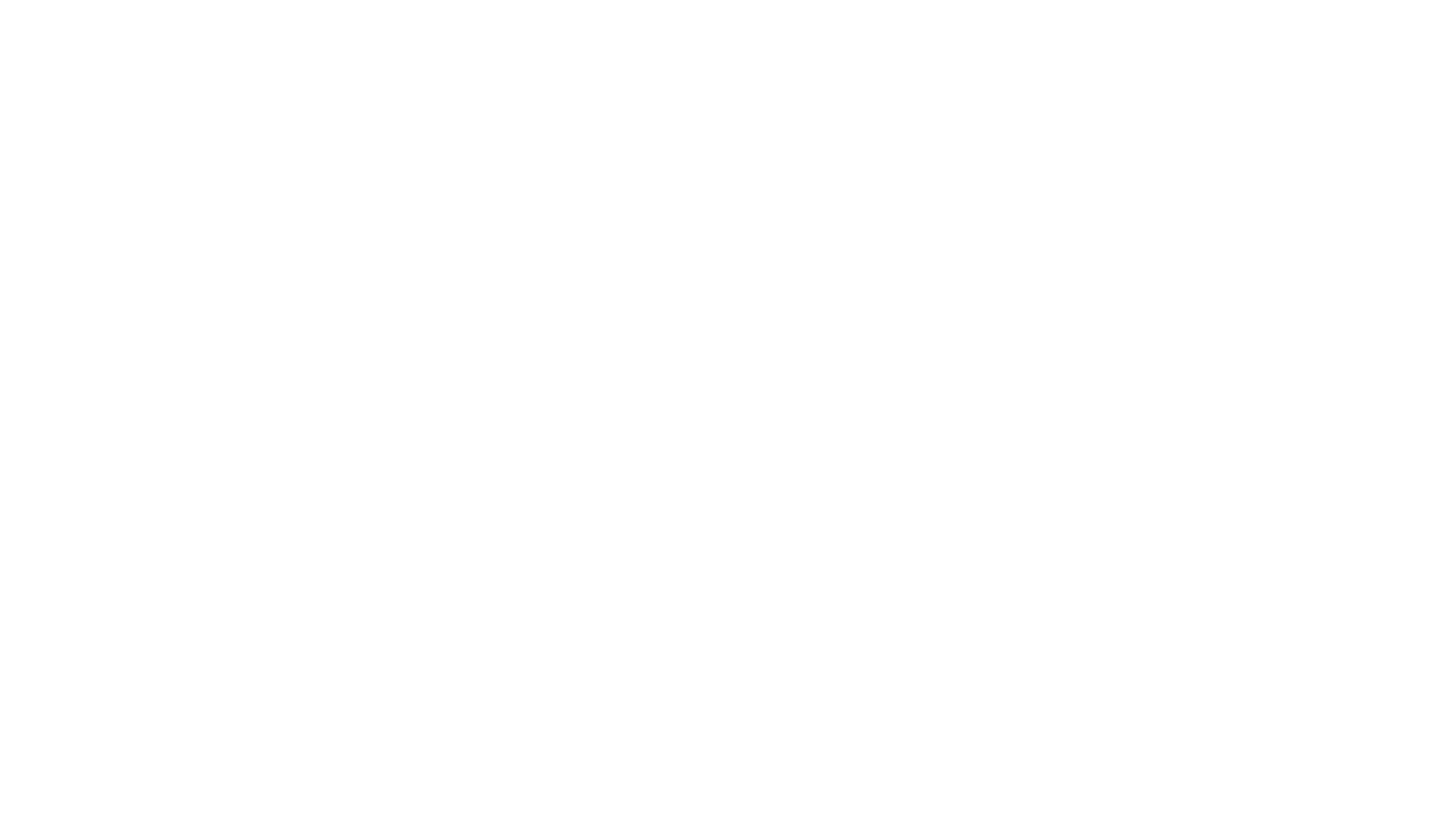

#

## Slide 4
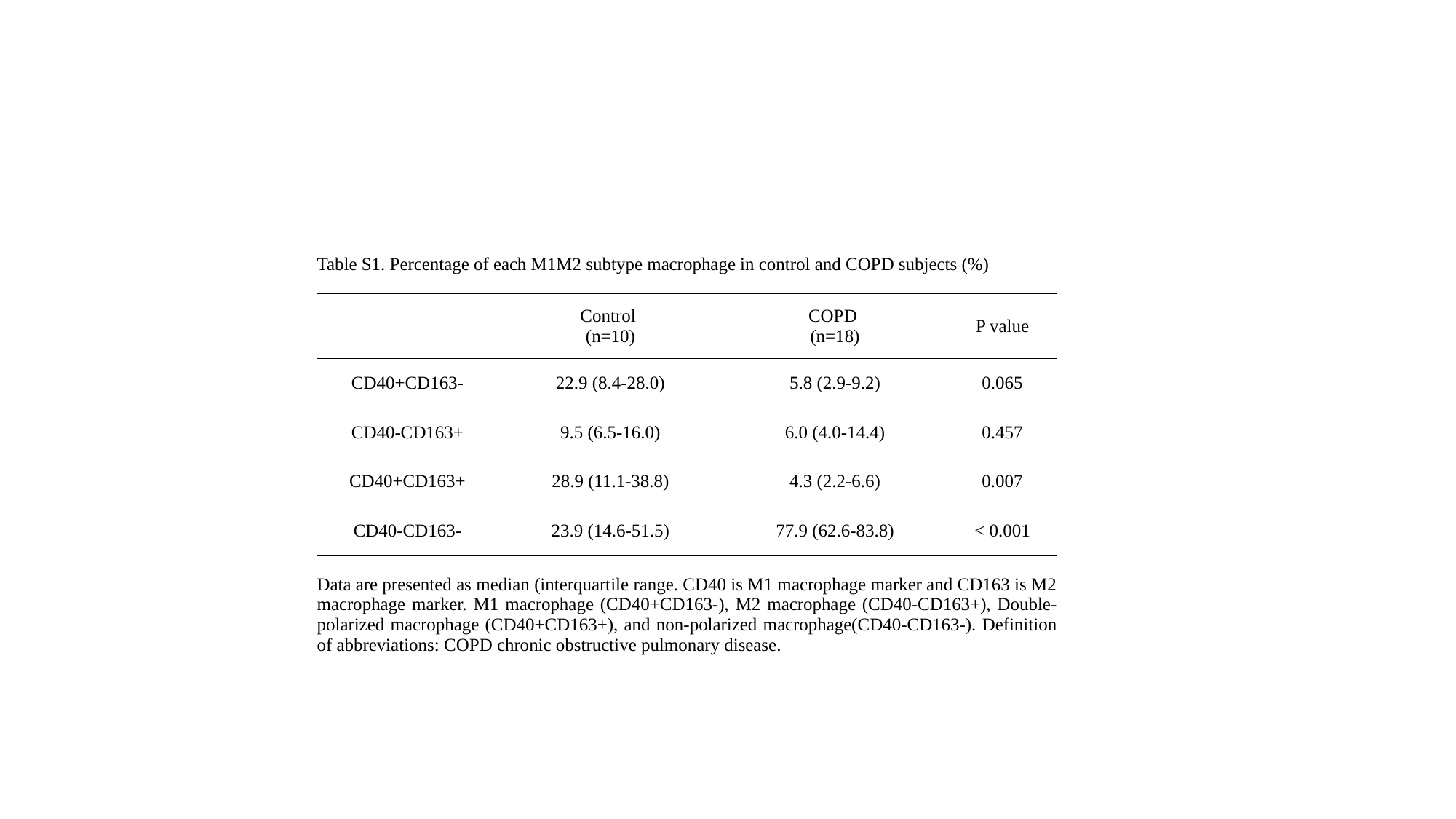

| Table S1. Percentage of each M1M2 subtype macrophage in control and COPD subjects (%) | | | |
| --- | --- | --- | --- |
| | Control (n=10) | COPD (n=18) | P value |
| CD40+CD163- | 22.9 (8.4-28.0) | 5.8 (2.9-9.2) | 0.065 |
| CD40-CD163+ | 9.5 (6.5-16.0) | 6.0 (4.0-14.4) | 0.457 |
| CD40+CD163+ | 28.9 (11.1-38.8) | 4.3 (2.2-6.6) | 0.007 |
| CD40-CD163- | 23.9 (14.6-51.5) | 77.9 (62.6-83.8) | < 0.001 |
| Data are presented as median (interquartile range. CD40 is M1 macrophage marker and CD163 is M2 macrophage marker. M1 macrophage (CD40+CD163-), M2 macrophage (CD40-CD163+), Double-polarized macrophage (CD40+CD163+), and non-polarized macrophage(CD40-CD163-). Definition of abbreviations: COPD chronic obstructive pulmonary disease. | | | |

## Slide 5
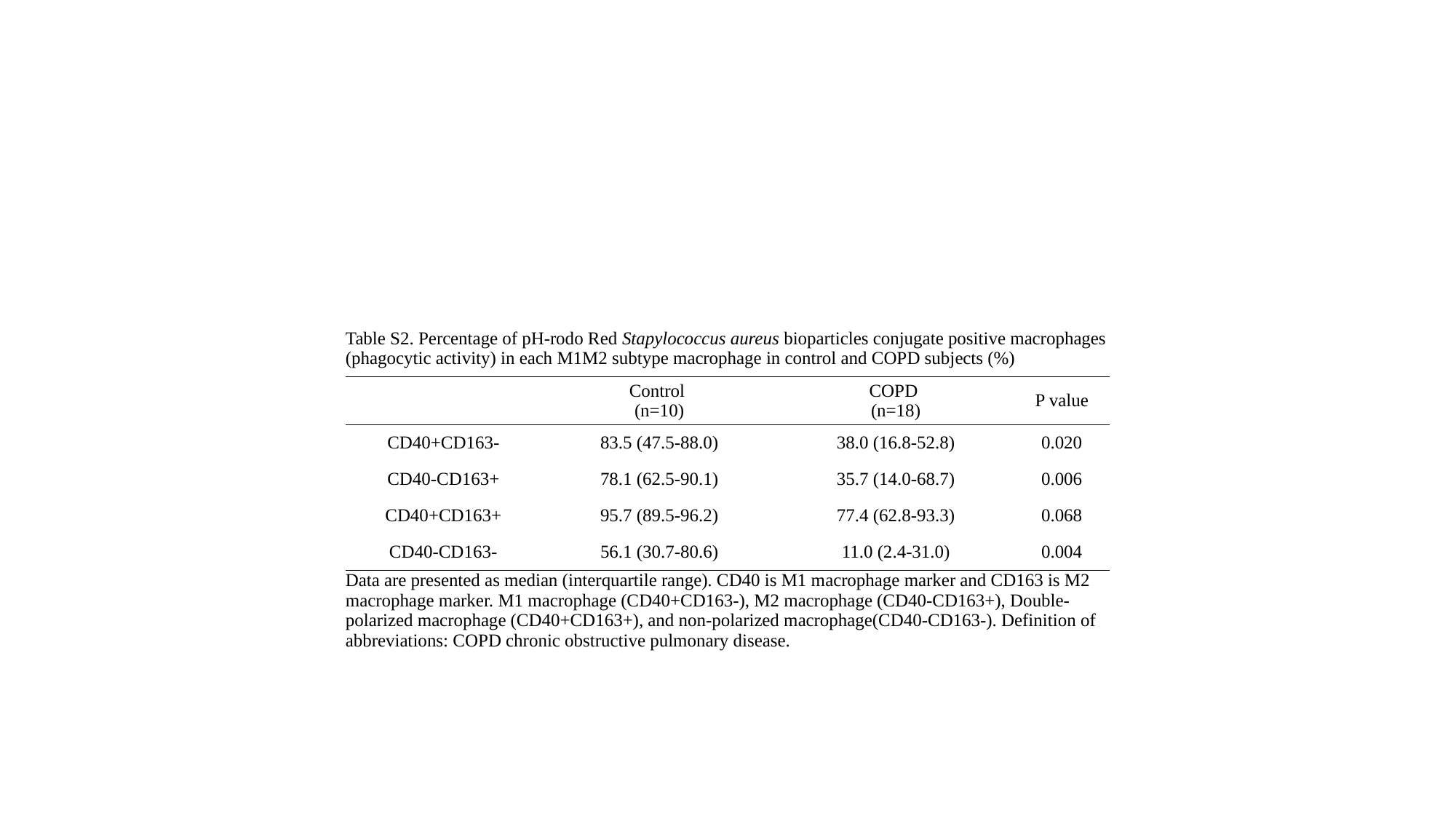

| Table S2. Percentage of pH-rodo Red Stapylococcus aureus bioparticles conjugate positive macrophages (phagocytic activity) in each M1M2 subtype macrophage in control and COPD subjects (%) | | | |
| --- | --- | --- | --- |
| | Control (n=10) | COPD (n=18) | P value |
| CD40+CD163- | 83.5 (47.5-88.0) | 38.0 (16.8-52.8) | 0.020 |
| CD40-CD163+ | 78.1 (62.5-90.1) | 35.7 (14.0-68.7) | 0.006 |
| CD40+CD163+ | 95.7 (89.5-96.2) | 77.4 (62.8-93.3) | 0.068 |
| CD40-CD163- | 56.1 (30.7-80.6) | 11.0 (2.4-31.0) | 0.004 |
| Data are presented as median (interquartile range). CD40 is M1 macrophage marker and CD163 is M2 macrophage marker. M1 macrophage (CD40+CD163-), M2 macrophage (CD40-CD163+), Double-polarized macrophage (CD40+CD163+), and non-polarized macrophage(CD40-CD163-). Definition of abbreviations: COPD chronic obstructive pulmonary disease. | | | |
